# Pinewood nematode induced changes in the assembly process of gallery microbiomes benefit its vector beetle’s development

**DOI:** 10.1128/spectrum.01412-24

**Published:** 2024-09-11

**Authors:** Bin Zhang, Yafei Ma, Wenzhao Duan, Qi Fan, Jianghua Sun

**Affiliations:** 1 College of Life Science/Hebei Basic Science Center for Biotic Interactions, Institute of Life Science and Green Development, Hebei University, Baoding, China; 2 State Key Laboratory of Integrated Management of Pest Insects and Rodents, Institute of Zoology, Chinese Academy of Sciences, Beijing, China; Chinese Academy of Sciences, Shanghai, China

**Keywords:** *Monochamus alternatus*, microbial assembly, gallery, pinewood nematode, beneficial microbiota

## Abstract

Microbiomes play crucial roles in insect adaptation, especially under stress such as pathogen invasion. Yet, how beneficial microbiomes assemble remains unclear. The wood-boring beetle *Monochamus alternatus*, a major pest and vector of the pine wilt disease (PWD) nematode, offers a unique model. We conducted controlled experiments using amplicon sequencing (16S rRNA and ITS) within galleries where beetles and microbes interact. PWD significantly altered bacterial and fungal communities, suggesting distinct assembly processes. Deterministic factors like priority effects, host selection, and microbial interactions shaped microbiome composition, distinguishing healthy from PWN-infected galleries. Actinobacteria, Firmicutes, and Ophiostomataceae emerged as potentially beneficial, aiding beetle’s development and pathogen resistance. This study unveils how nematode-induced changes in gallery microbiomes influence beetle’s development, shedding light on microbiome assembly amid insect-pathogen interactions. Insights gleaned enhance understanding of PWD spread and suggest novel management strategies via microbiome manipulation.

**IMPORTANCE:**

This study explores the assembly process of gallery microbiomes associated with a wood-boring beetles, *Monochamus alternatus*, a vector of the pine wilt disease (PWD). By conducting controlled comparison experiments and employing amplicon approaches, the study reveals significant changes in taxonomic composition and functional adaptation of bacterial and fungal communities induced by PWD. It identifies deterministic processes, including priority effects, host selection, and microbial interactions, as major drivers in microbiome assembly. Additionally, the study highlights the presence of potentially beneficial microbes such as Actinobacteria, Firmicutes, and Ophiostomataceae, which could enhance beetle development and resistance to pathogens. These findings shed light on the intricate interplay among insects, microbiomes, and pathogens, contributing to a deeper understanding of PWD prevalence and suggesting innovative management strategies through microbiome manipulation.

## INTRODUCTION

Microbiomes co-evolve with their insect hosts as a holobiont, playing a crucial role in benefiting the hosts in numerous aspects. For example, the microbes of insect gut and their surface may potentially contribute to supplementing nutrition, detoxifying defensive compounds, resisting pathogens, enhancing immune homeostasis, promoting invasion, manipulating behavior, and physiology (1–7). In addition to affecting the physiological and behavioral phenotypes, differences in structure and diversity of microbiome may also have substantial impacts on host phenotypic plasticity and functional extension within populations, especially under ecological pressures, thus promoting the utilization of novel ecological niches and contributing to the evolution and adaptation of the holobiont of host and microbiome (8, 9). Consequently, understanding the force and processes by which insects acquire their beneficial microbiomes is not only one of the central goals of host microbes research (10), but also have potentials to develop environmentally sustainable strategies for pest and disease management via microbiome manipulation (11). However, the fundamental ecological process that governs the assembly of insect beneficial microbiome is still elusive and highly underexplored.

The assembly of microbiomes is a complex and dynamic process shaped by multiple mechanisms (12, 13). Generally, microbial community assembly is influenced by stochastic and/or deterministic processes (14). The traditional niche-based theory asserts that deterministic processes are associated with ecological selection and are determined by a variety of biotic and abiotic factors, such as host phylogeny, developmental stage and genetic background, species interaction and successional dynamics (15, 16), geographical location, temperature, and PH (10, 17). In contrast, stochastic processes are referred to as ecological processes that generate community diversity patterns through random processes, such as birth, death, colonization, immigration, speciation, and dispersal limitations (18). Although a plethora of studies support that niche-based and neutral processes are jointly or separately responsible for microbial community assembly (19–21), elucidating the relative roles of niche and neutral processes in community assembly remains an ongoing challenge. Another challenge in disentangling the assembly processes of microbiome is evaluating the origination and transmission route of core microbes in different continua, such as soil-plant-insect (22), multiple tropic network (23), as well as over time. Often these microbes are either horizontally sourced from adjacent local environmental pools of microbes or vertically originated from the mother of the host insects (24). However, the extent to which and the dynamics of core microbes over developmental time are still elusive. To comprehensively understand the transmission and assembly process of microbiome, we should not only focus on an individual host but also investigate the microbial communities in its surrounding environment (25).

Wood-boring insects are widely recognized for their symbiotic relationships with microbes, holding significant economic and ecological importance for facilitating nutrient cycling and forest succession processes (26–30). The galleries they created serve as unique habitats akin to gardens, furnishing essential resources and microenvironments crucial for the insects' survival and reproduction. Much like gardeners, wood-boring insects engineer their galleries, recruiting and enriching a suite of beneficial microbiomes to fulfill their needs, such as acquiring nutrients and resisting pathogens. A burgeoning body of research has highlighted the gallery as a critical zone for the holobiont of wood-boring insects and their associated microbiomes (31, 32). For instance, the distinctive features of hidden and enclosed spaces within galleries not only facilitate the faithful transmission of core microbes, whether vertically or horizontally, but also serve as a means to selectively filter the microbiome for the insect host (22, 23, 33). Given these characteristics and functions, the gallery provides an ideal system for investigating the assembly mechanisms of beneficial microbiomes, which remain largely unexplored.

The Japanese sawyer beetle, *Monochamus alternatus*, is not only a serious wood-boring pest in itself, but is also the main vector of the invasive pinewood nematode (PWN), *Bursaphelenchus xylophilus*, which is the causal agent of pine wilt disease (PWD) and poses a serious threat to pine forests globally (34). A plethora of studies suggested that microbes play a non-negligible role in the prevalence of both this vector beetle and PWD (35, 36). However, these studies primarily focused on isolated partners or environments within this complex, thereby failing to fully capture the effects of their interactions on the microbial community. For example, pines infected with PWD have been shown to harbor a divergent bacterial and fungal communities compared to healthy trees (33, 37–39). Yet, whether these changes in the microbiome induced by PWD affect the fitness of vector beetles is not well investigated. Additionally, the *M. alternatus* beetle exhibits a strong oviposition preference to the weakened pine trees infected with PWN (40), a preference critical for the life cycle synchronization and evolution of *M. alternatus* and PWN symbiosis. This preference might result from the low defensive ability of the weakened trees. However, they also harbor an obvious harsh condition with restricted available nutrients but abundant pathogens, threatening the survival and development of their offspring (41). An increasing number of studies have shown that beneficial microbiomes in the microhabitat usually aid in the defense against pathogens and overcoming nutritional limitations (6, 42). For instance, abundant blue-staining fungi symbiotic with the vector beetle were found around its galleries, among which *Sporothrix* sp. significantly promoted the growth and development of *M. alternatus* (35). Therefore, deciphering the structure and the assembly process of beneficial microbiomes within galleries is crucial for elucidating the adaptation mechanisms of this vector beetle to the PWD-infested trees.

In this study, we hypothesized that PWN could induce changes in the assembly process and enrich more beneficial microbiome in galleries, sourced either vertically from the mother beetle or horizontally from pine trees infected with PWD. This divergence in microbiome then promotes the fitness and stress adaptation of the vector beetle’s offspring. To test this hypothesis, we explored the taxonomic and functional differences between the microbiomes of healthy and PWD-infected pine trees using 16S rRNA and ITS sequencing. Additionally, we assessed the survival rate and weight of the vector beetle’s offspring over the larval development time series. To investigate the assembly mechanism and potential sources, we conducted neutral model test, tracking analysis, and co-occurrence network analysis of healthy and diseased gallery microbiomes. Furthermore, we identified the core beneficial microbiomes enriched and deleted in healthy and diseased galleries, respectively. Understanding the assembly process of beneficial microbiomes in such systems would not only enhance our comprehension of the intricated interactions among pathogens, vector insects, and their microbiomes, which contribute to the prevalence of PWD, but also facilitate the development of biocontrol methods for pests and diseases.

## RESULTS

### PWD induced changes in composition and diversity of bacterial and fungal communities in both source and assembly samples

**Fig 1:**
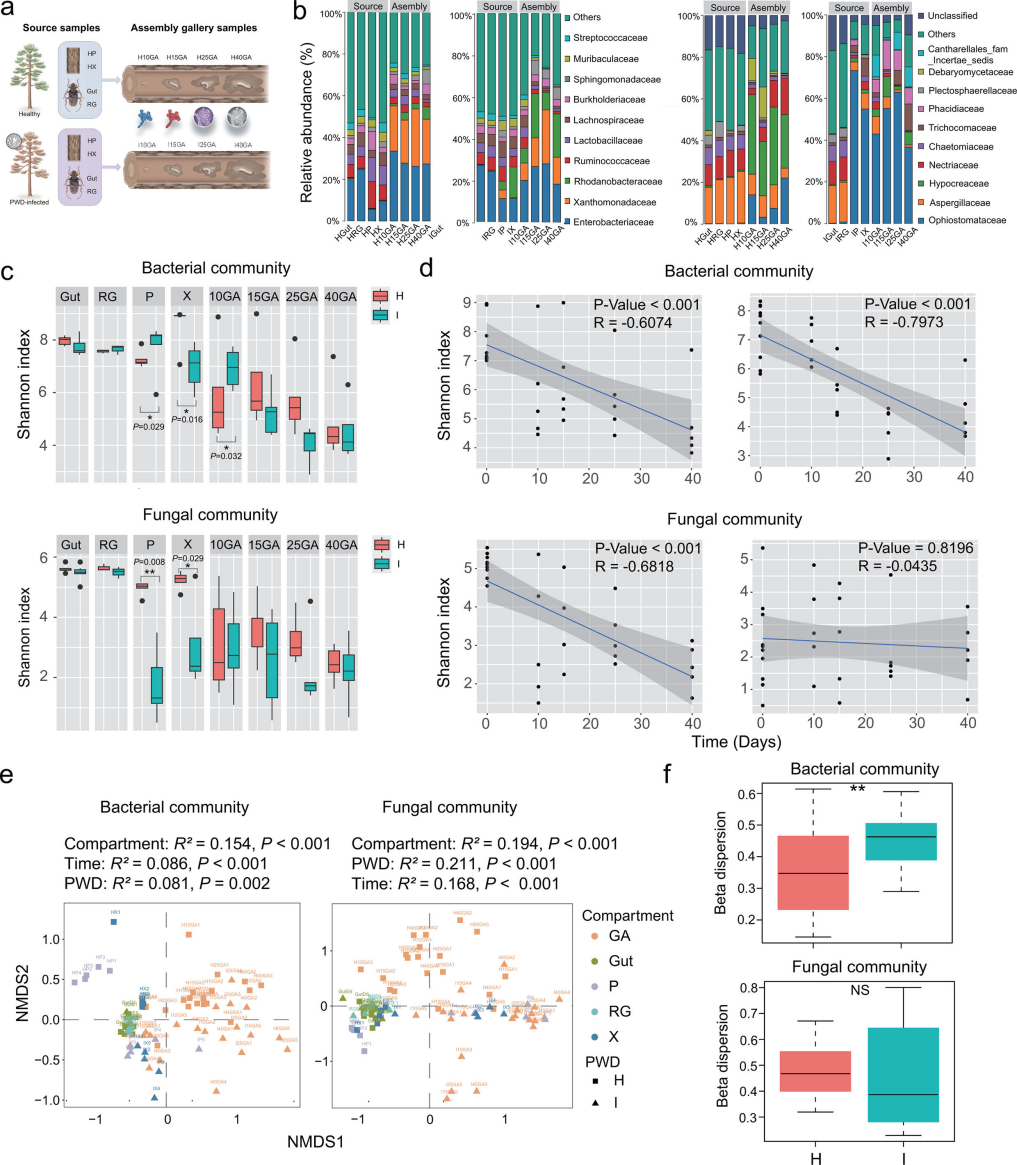
PWD induced changes in assembly of bacterial and fungal communities over developmental time. (**a**) Sampling and experimental procedure. Samples were categorized into three groups: (i) source beetle samples, comprising gut (Gut) and reproductive glands (RG) samples from female beetles; (ii) source pine samples, consisting of xylem (HX and IX) and phloem (HP and IP) extracted from pine tree logs; (iii) assembly gallery samples, collected over a developmental time series from galleries (H10GA, H15GA, H25GA, and H40GA for healthy galleries, I10GA, I15GA, I25GA, and I40GA for PWD-infected galleries). (**b**) The composition of bacterial (left) and fungal community (right) of top 10 families in relative abundances in both source and assembly samples from healthy and PWD-infected treatments, respectively. (**c**) The changes of Shannon diversity index of bacterial (above) and fungal (bottom) community during the microbiome assembly process from both healthy (H) and PWD-infected (I) logs. Data points corresponding to samples. Wilcoxon rank-sum test, ns, not significant, *P < 0.05, **P < 0.01, ***P < 0.001. (**d**) Shannon diversity is plotted against development time for bacterial and fungal sampled from healthy (H, left) and PWD-infected (I, right) logs. Blue lines indicate the linear mixed-effects regression of diversity on time. Shading indicates the 95% confidence interval (CI). (**e**) Non-metric multi-dimensional scaling (NMDS) ordinations of Bray–Cutis dissimilarity matrices with permutational analysis of variance (PERMANOVA), showing significant association of the bacterial (left) and fungal (right) community composition with the following factors ranked in order of importance: compartment (*R*^2^ = 0.154), time (*R*^2^ = 0.086), and PWD (*R*^2^ = 0.081) for bacteria; and PWD (*R*^2^ = 0.211), compartment (*R*^2^ = 0.194), and time (*R*^2^ = 0.168) for fungi, respectively. (**f**) Beta-dispersion analysis based on Bray–Cutis dissimilarity for bacterial and fungal communities. The difference significance between healthy and PWD-infected treatments was tested using the Wilcoxon rank-sum test.

After quality and chimeric sequence filtering, we obtained 5,903,187 bacterial 16S rRNA and 5,564,470 fungal ITS high-quality reads from 80 samples. These reads were sorted into 1,608 bacterial OTUs (Dataset S1) and 1,369 fungal OTUs (Dataset S2). For both the source and assembly microbial communities, the taxonomic composition at the family level differed significantly between healthy and PWD-infected logs for both bacterial and fungal communities (Fig. 1b).

In the bacterial source community, PWD-infected xylem (IX) and phloem (IP) were dominated by Enterobacteriaceae, Rhodanobacteraceae, Ruminococcaceae, and Lachnospiraceae, accounting for 37.0% and 30.2% of sequences, respectively. Healthy xylem (HX) and phloem (HP) were characterized by Ruminococcaceae, Lactobacillaceae, Enterobacteriaceae, and Burkholderiaceae, comprising 28.6% and 38.9% of sequences, respectively. During bacterial assembly, PWD-infected galleries showed predominant families such as Enterobacteriaceae, Xanthomonadaceae, and Rhodanobacteraceae across developmental stages (I10GA to I40GA), with relative abundances ranging from 35.5% to 62.4%. Conversely, healthy galleries exhibited dominant Enterobacteriaceae, and Xanthomonadaceae, with relative abundances from 48.3% to 55.1% (Fig. 1b; Table S2). In the fungal source community, PWD-infected xylem (IX) and phloem (IP) were primarily composed of Ophiostomataceae, Aspergillaceae and Trichocomaceae, representing 71.2% and 80.9% of sequences, respectively. Healthy xylem (HX) and phloem (HP) featured Aspergillaceae, Nectriaceae, and Chaetomiaceae, comprising 41.6% and 41.5% of sequences, respectively. During fungal assembly, PWD-infected galleries displayed major families such as Ophiostomataceae, Trichocomaceae, and Cantharellales_fam_Incertae_sedis, with relative abundances from 47.3% to 72.5%. Healthy galleries showed dominant families like Hypocreaceae, Aspergillaceae, Ophiostomataceae, and Nectriaceae, with relative abundances from 46.4% to 70.0% (Fig. 1b; Table S3). Moreover, in both bacterial and fungal assembly communities, the top 10 families showed significantly higher relative abundances compared to source communities for both healthy and PWD-infected samples, indicating pronounced shifts during gallery development (Fig. 1b; Table S3).

The alpha diversity, measured by the Shannon index, of bacterial and fungal communities in both xylem and phloem of source trees showed significant changes following PWD infection (Fig. 1c). In bacterial communities, PWD infection increased Shannon diversity in phloem (Wilcoxon rank-sum test: *P* = 0.029) but decreased it in xylem (Wilcoxon rank-sum test: *P* = 0.016) compared to healthy communities. Conversely, fungal communities exhibited decreased Shannon diversity in both phloem (Wilcoxon rank-sum test: *P* = 0.008) and xylem (Wilcoxon rank-sum test: *P* = 0.029) due to PWD infection. During assembly, alpha diversity significantly decreased with development in both bacterial (Linear regression: Healthy: *R* = −0.607, *P* < 0.001; PWD-infected: *R* = −0.797, *P* < 0.001) and fungal communities (Linear regression: Healthy: *R* = −0.682, *P* < 0.001), except for PWD-infected fungal communities where diversity remained stable (Linear regression: PWD-infected: *R* = −0.044, *P* = 0.820) (Fig. 1d).

We then examined the relative contributions of the factors in terms of compartment, PWD, and time to the assembly process and shaping the gallery microbiome (Dataset S3). NMDS ordinations and PERMANOVA analysis revealed that the bacterial gallery microbiome was most significantly influenced by the compartment (*R*^2^ = 0.154, *P* < 0.001), followed by time (*R*^2^ = 0.086, *P* < 0.001), and PWD (*R*^2^ = 0.081, *P* = 0.002) (Fig. 1e; Table S4). In contrast, the fungal community exhibited the highest proportion of variation explained by PWD (*R*^2^ = 0.211, *P* < 0.001), followed by compartment (*R*^2^ = 0.194, *P* < 0.001) and time (*R*^2^ = 0.168, *P* < 0.001) (Fig. 1e; Table S4). To determine the variation of microbiome, we conducted beta dispersion analysis using Bray–Curtis dissimilarity, which revealed that the bacterial community of PWD-infected samples exhibited greater variability compared to healthy ones (*P* = 0.01), while the fungal community showed no significant difference in variation between healthy and PWD-infected galleries (*P* = 0.586) (Fig. 1f).

### PWD induced alternations in taxonomic biomarkers of gallery microbiome and larval beetle fitness

**Fig 2:**
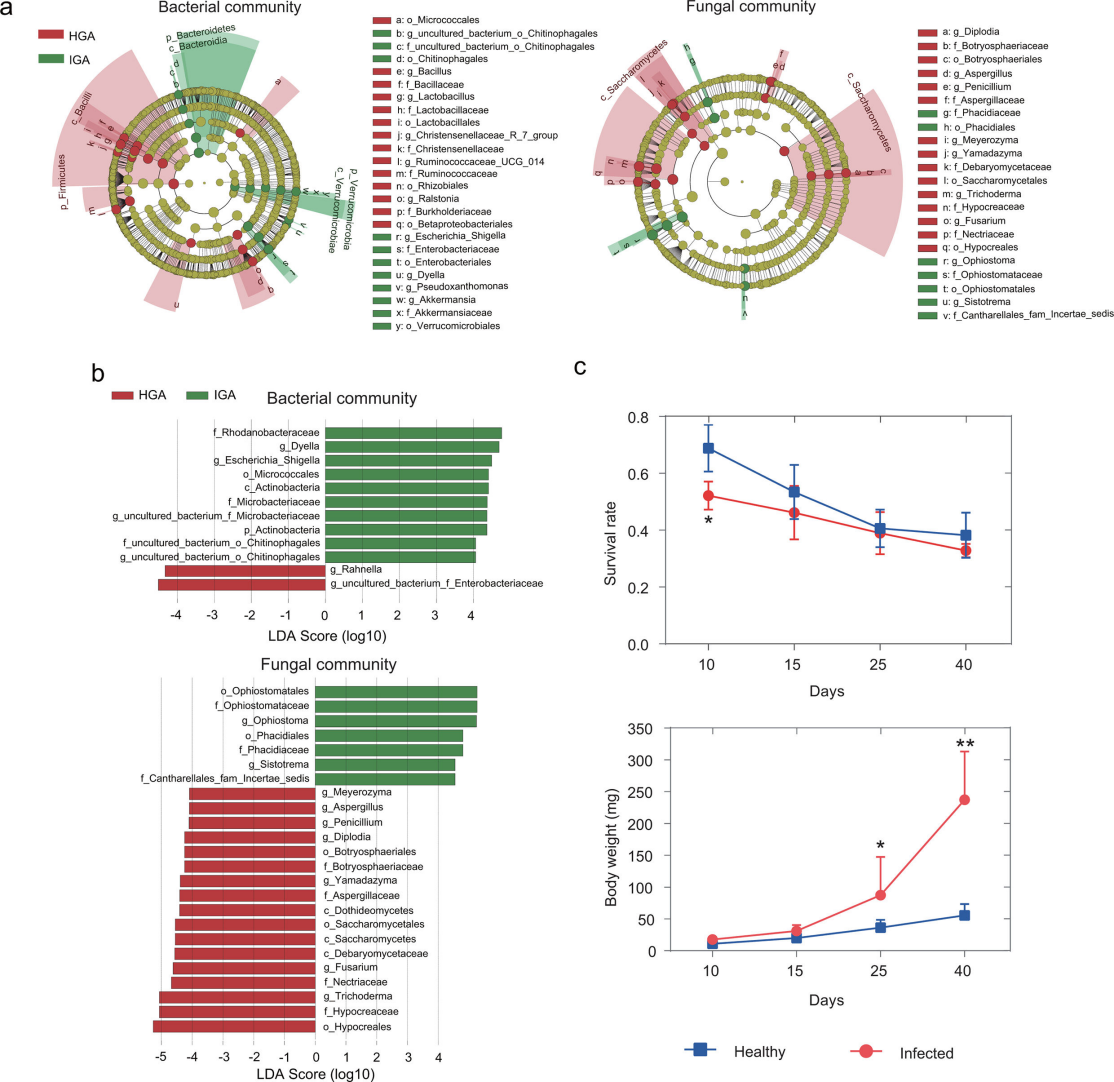
PWD induced changes in functions of gallery microbiome and fitness of larval beetle. (**a**) Cladogram representation of differentially abundant taxonomic clades in bacterial and fungal communities of both healthy and PWD-infected galleries. In the cladogram, circles radiate from the center outwards, depicting taxonomic levels from phylum to genus. Each small circle represents a classification at various levels, with the diameter of the circles proportional to their relative abundance. Classifications with no significant difference are marked in yellow, while red and green signify healthy (HGA) and PWD-infected (IGA) galleries, respectively. Differentially abundant taxa between healthy and PWD-infected galleries, revealed by linear discriminant analysis effect size (LEfSe) analysis, with the bacteria of *P* < 0.05 and LDA score >4. (**b**) LEfSe analysis showing the indicator taxa in bacterial (above) and fungal (bottom) community associated either with healthy (HGA) or infected (IGA) galleries. The taxonomic levels are from family to genus with *P* < 0.05 and LDA score threshold 4. (**c**) Comparison of changes in survival rate (left) and body weight (right) of larva from healthy (HGA) or infected (IGA) logs across 40 days’ development. Wilcoxon rank-sum test, ns, not significant, *P < 0.05, **P < 0.01, ***P < 0.001.

To discern the differentially abundant taxa within assembled microbial communities between healthy (HGA) and PWD-infected (IGA) galleries, we conducted linear discriminant analysis effect size (LEfSe) analysis. The LEfSe Cladogram highlighted significant differences, with the bacterial phylum Firmicutes clade being notably higher, while the phylum Bacteroidetes and Verrucomicrobia clades were significantly lower in healthy galleries compared to those in PWD-infected galleries. Similarly, the fungal order Ophiostomatales and Phacidiales, as well as the family Cantharellales_fam_Incertae_sedis clades exhibited significantly higher abundance, while the class Saccharomycetes and Saccharomycetes clades were notably lower in PWD-infected galleries (Fig. 2a). Through further employing LEfSe LDA analysis, we identified taxonomic biomarkers distinguishing healthy and PWD-infected galleries. Specifically, 10 bacterial and 7 fungal taxa displayed significantly higher abundance, whereas 2 bacterial and 17 fungal taxa exhibited significantly lower abundance in PWD-infected galleries compared to healthy ones (LEfSe analysis, *P* < 0.05, LDA score >4) (Fig. 2b).

To further corroborate the functional divergence of microbial communities between HGA and IGA, we examined differences in larval fitness, including survival rate and body weight, over development time series. The results showed that the survival rate of offspring larva from both healthy and PWD-infected galleries decreased over time. Specifically, the survival rate exhibited significantly differences at 10 days, but there is no significant difference between IGA and HGA after 15 days (Wilcoxon rank-sum test: 10 days, *P* = 0.016; 15 days, *P* = 0.078; 25 days, *P* = 0.486; 40 days, *P* = 0.343; Fig. 2c). In contrast, the body weight of offspring larva from both healthy and PWD-infected group increased during the development, with a significant difference observed after 25 days. The larva from PWD-infected galleries group showed significantly higher body weight compared to those from healthy ones (Wilcoxon rank-sum test: 10 days, *P* = 0.056; 15 days, *P* = 0.095; 25d, *P* = 0.016; 40d, *P* = 0.008; Fig. 2c).

### Assembly mechanism of gallery microbiome

**Fig 3:**
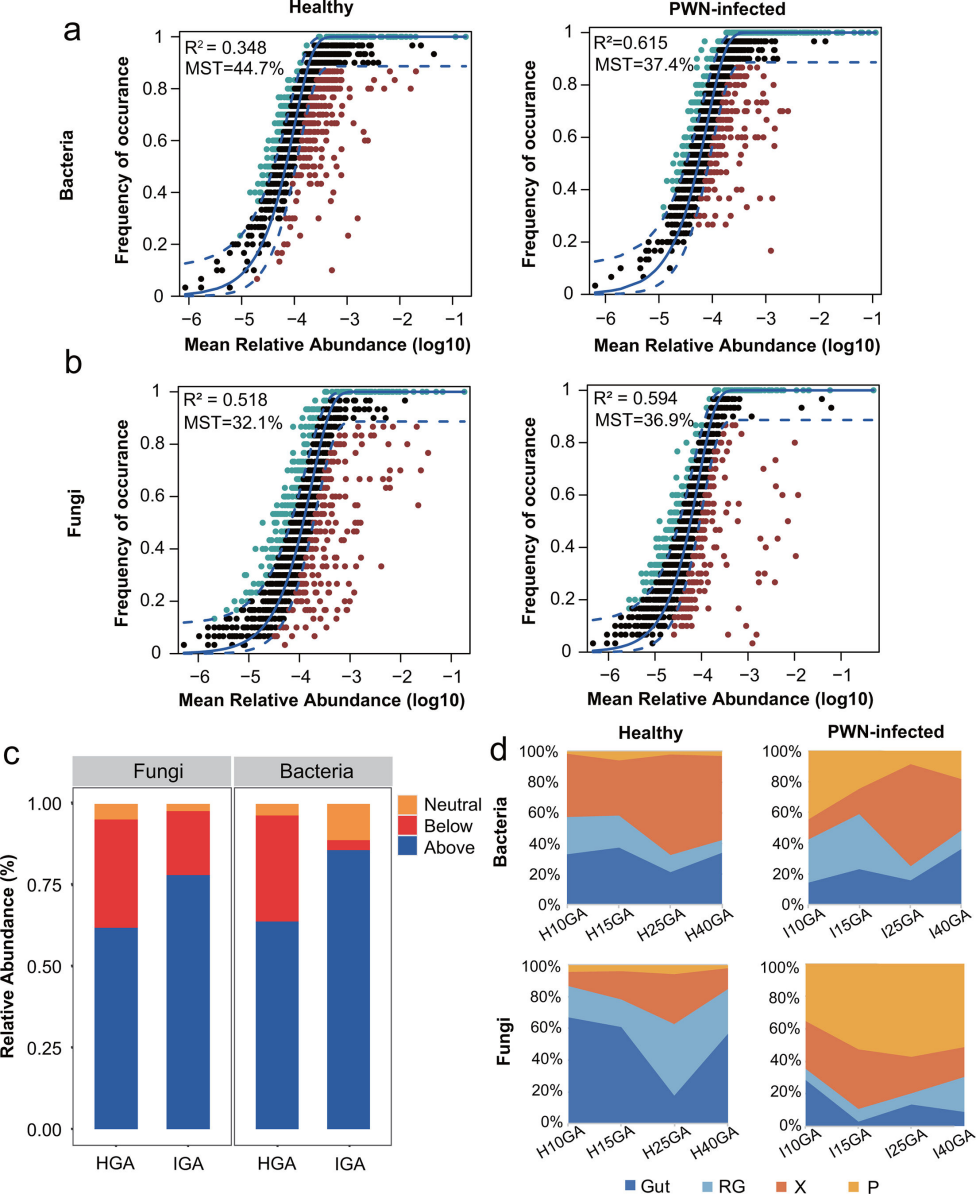
Difference in assembly mechanism of gallery microbiome between healthy and PWD-infected galleries. (**a and b**) Sloan neutral model for bacterial (**a**) and fungal (**b**) communities for assembly samples from healthy (left) and PWD-infected (right) logs. Blue dashed lines represent 95% confidence intervals around the model prediction, with OTUs fitting the model depicted in black. Each OTU is represented by a data point. OTUs occurring more frequently than predicted by the model are colored blue, while those occurring less frequently are colored red. The *R*² values, indicating the fit to the neutral assembly process, and Nm values, estimating migration rate, are indicated for each prediction. (**c**) The cumulative relative abundance distribution of three types of OTUs of the neutral model for bacterial and fungal communites in healthy and PWD-infected treatment, respectively. (**d**) Source tracking analysis of gallery microbiota using FEAST, based on the Bayesian approach showing the difference in potential source of bacterial (above) and fungal (bottom) communities across development time points (10, 15, 25, and 40 days) for samples collected from healthy (left) and PWD-infected (right) logs, respectively. Gut (dark blue) and reproductive gland (RG, light blue) from the mated female, as well as xylem (X, orange) and phloem (P, yellow) from egg-free logs were set as “source,” while the gallery samples were designated as “sinks.”

To explore the assembly mechanisms of microbiomes in healthy and PWD-infected galleries, an analysis of neutral community model (NCM) with modified stochasticity ratio (MST) and test of null model with β-nearest taxon index (βNTI) were implemented, respectively. The NCM results indicated that the quality of fit was higher for PWD-infected galleries than for healthy galleries in both bacterial and fungal communities (for bacteria, HGA: *R*^2^ = 0.348, IGA: *R*^2^ = 0.615; and for fungi, HGA: *R*^2^ = 0.518, IGA: *R*^2^ = 0.594, respectively) (Fig. 3a and b). The MST value suggested that the microbial communities in both healthy and PWD-infected galleries were more strongly driven by deterministic assembly processes (MST in bacteria: HGA: 44.7%, IGA: 37.4%; MST in fungi: HGA: 32.1%, IGA: 36.9%, respectively) (Fig. 3a and b). Notably, the cumulative relative abundances of the OTUs below prediction accounted for much higher proportion in healthy galleries compared to PWD-infected galleries (for bacteria: HGA, 32.58% and IGA: 3.04%; for fungi: HGA, 33.27% and IGA: 19.66%, respectively) (Fig. 3c; Table S5). Similarly, null model tests and βNTI results indicated that deterministic factors (i.e., selection) predominantly govern the assembly process of the gallery microbiome (βNTI > 2) (Fig. S1).

To characterize the sources of the assembled microbiome in galleries, we performed source tracking analysis over developmental time. Fast expectation-maximization microbial source tracking (FEAST) analysis revealed that bacterial communities of healthy and PWD-infected galleries plausibly originated from the xylem and phloem microbiota, with this origin showing an increasing tendency along with larval development (Fig. 3d). similarly, a large proportion of fungal communities of PWD-infected galleries originated from the pine trees (Xylem and phloem). In contrast, the fungal community of healthy galleries primarily originated from the beetle (gut and reproductive gland) (Fig. 3d).

### Co-occurrence network

To explore how PWD affects the co-occurrence patterns of the gallery microbiome from source to assembled samples, we analyzed the bacterial-bacterial and fungal-fungal intra-kingdom networks as well as the bacterial-fungal interkingdom networks, and compared changes in networks of microbiomes from source tree to assembled galleries between healthy and PWD-infected treatments. Metrics, such as the number of edges, average path length, and clustering coefficient, represent the complexity of the network and the strength of interactions among microorganisms. Additionally, the number of positive and negative edges indicated stability.

**Fig 4:**
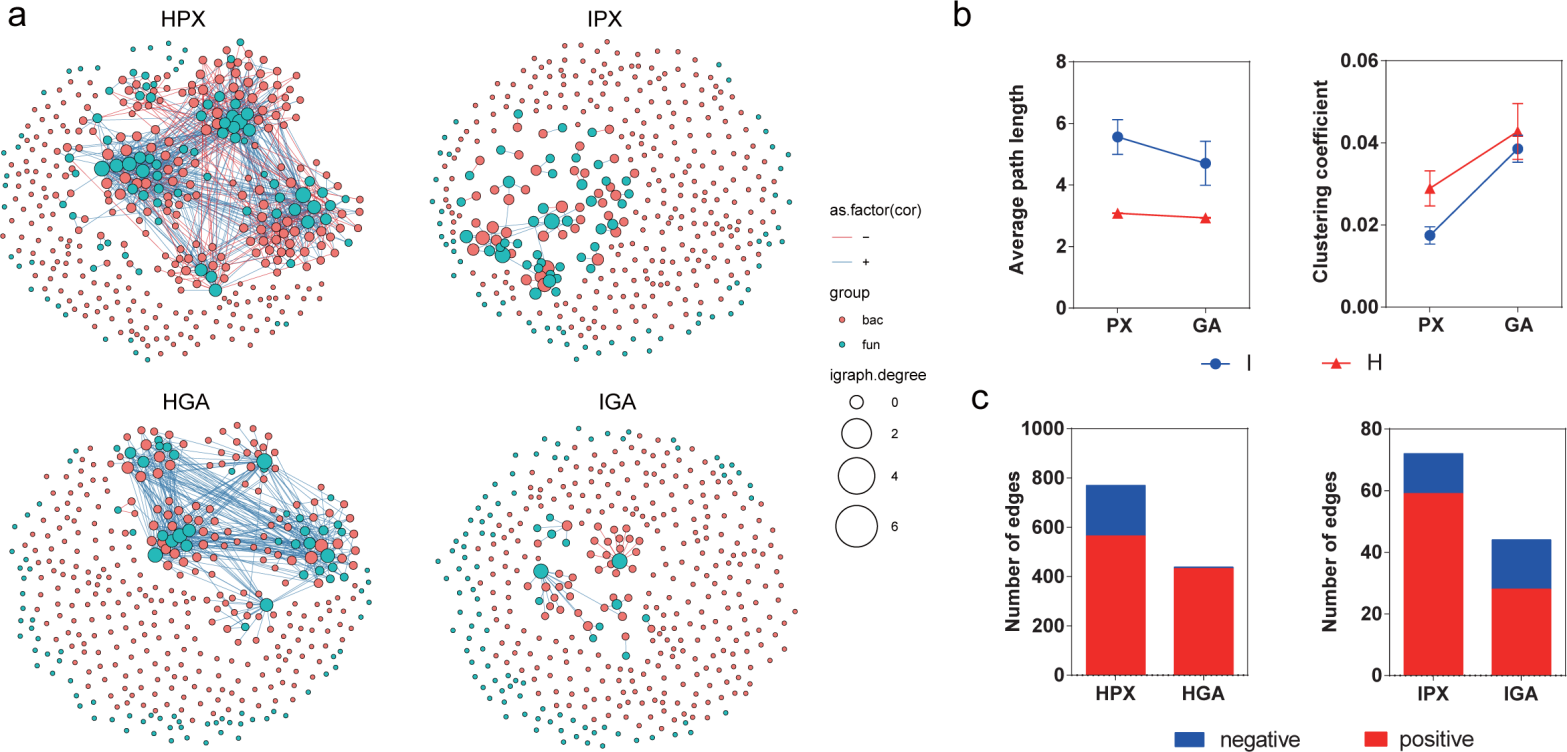
Changes of interkingdom co-occurrence networks between healthy and PWD-infected galleries. (**a**) Interkingdom co-occurrence networks showing a higher complexity of healthy logs compared to PWD-infected logs, evident in both source samples (HPX vs. IPX) and gallery assembly samples (HGA vs. IGA). The top 150 ITS and top 200 16S rRNA OTUs, based on relative abundance, were selected for network analysis. Nodes are color-coded according to bacterial (red) and fungal (blue) OTUs, with node size indicating the degree of connection. Edge color represents positive (green) and negative (red) correlations. These networks were constructed using corBionetwork in the ggClusterNet package of R software (Version 4.0.5), with a connection threshold of *R* = 0.75 and a significance level of *P* < 0.05. (**b**) Comparison of the values of average path length and clustering coefficient for gallery samples in both healthy (H) and PWD-infected (I) treatments. (**c**) The number of bacterial–fungal correlations in the healthy and diseased networks, indicating a higher number and percentage of positive correlations in HGA compared to IGA.

The inter-kingdom network analysis revealed that the complexity and stability of microbiome networks from both healthy and PWD-infected treatments decreased from source to assembled samples. Additionally, the microbiome interaction in healthy galleries exhibited higher complexity than of the PWD-infected galleries, in both source and assembly samples. Specifically, the average path length of the PWD-infected networks was much higher than that of the healthy ones, and had a decreasing tendency from the source to assembled samples in both healthy (PX: 3.079 ± 0.011; GA: 2.932 ± 0.015) and PWD-infected (PX: 5.558 ± 0.563; GA: 4.703 ± 0.714) treatments. Conversely, the clustering coefficient of PWD-infected networks was lower than healthy ones, exhibiting an increasing tendency from the source to assembled samples in both healthy (PX: 0.029 ± 0.004; GA: 0.043 ± 0.007) and PWD-infected (PX: 0.017 ± 0.021; GA: 0.039 ± 0.033) treatments (Fig. 4a and b; Table S6). Moreover, the total number of edges in healthy co-occurrence networks was much higher than that in PWD-infected ones. Meanwhile, the number of edges decreased sharply from source to assembled microbiome for both healthy (770 for PX to 438 for GA) and PWD-infected (72 for PX to 44 for GA) treatments. The ratio of the number of negative to positive edges in healthy galleries showed a much larger decrease compared to PWD-infected galleries, from source to assembled samples, suggesting a divergence in microbial network destabilization between healthy and diseased galleries (Fig. 4c; Table S6).

In the bacterial-bacterial networks, we recorded that a larger increase in the complexity, and a smaller decrease in the stability have PWD-infected treatments compared to PWD-infected ones, from source trees to assembly galleries. Specifically, the average path length and clustering coefficient displayed a larger decrease and increase, respectively, in PWD-infected treatments (average path length: from 1.902 for IPX to 1.706 for IGA; clustering coefficient: from 0.141 for IPX to 0.293 for IGA) than in healthy ones (average path length: from 1.781 for HPX to 1.747 for HGA; clustering coefficient: from 0.219 for HPX to 0.253 for HGA). Furthermore, the decrease in the proportion of negative edges was much larger in healthy treatments (from 23.8% in HPX to 3.5% in HGA) than in PWD-infected ones (from 20.6% in IPX to 3.9% in IGA), from source to assembled samples (Fig. S2; Table S6).

In the fungal-fungal intra-kingdom networks, we observed similar patterns where the average path length was much lower and clustering coefficient much higher in PWD-infected treatments compared to healthy ones. However, the average path length exhibited a decreasing tendency, and the clustering coefficient showed an increasing tendency from the source to assembled communities in both healthy (average path length: from 1.750 for HPX to 1.640 for HGA; clustering coefficient: from 0.250 for HPX to 0.360 for HGA) and PWD-infected treatments (average path length: from 1.614 for IPX to 1.506 for IGA; clustering coefficient: from 0.386 for IPX to 0.494 for IGA). Moreover, the decrease in the proportion of negative edges was much larger in healthy communities (from 34.5% in HPX to 0.1% in HGA) than that in PWD-infected communities (from 2.0% in IPX to 0.1% in IGA) (Fig. S3; Table S6).

### Core microbiome and enriched beneficial taxa

**Fig 5:**
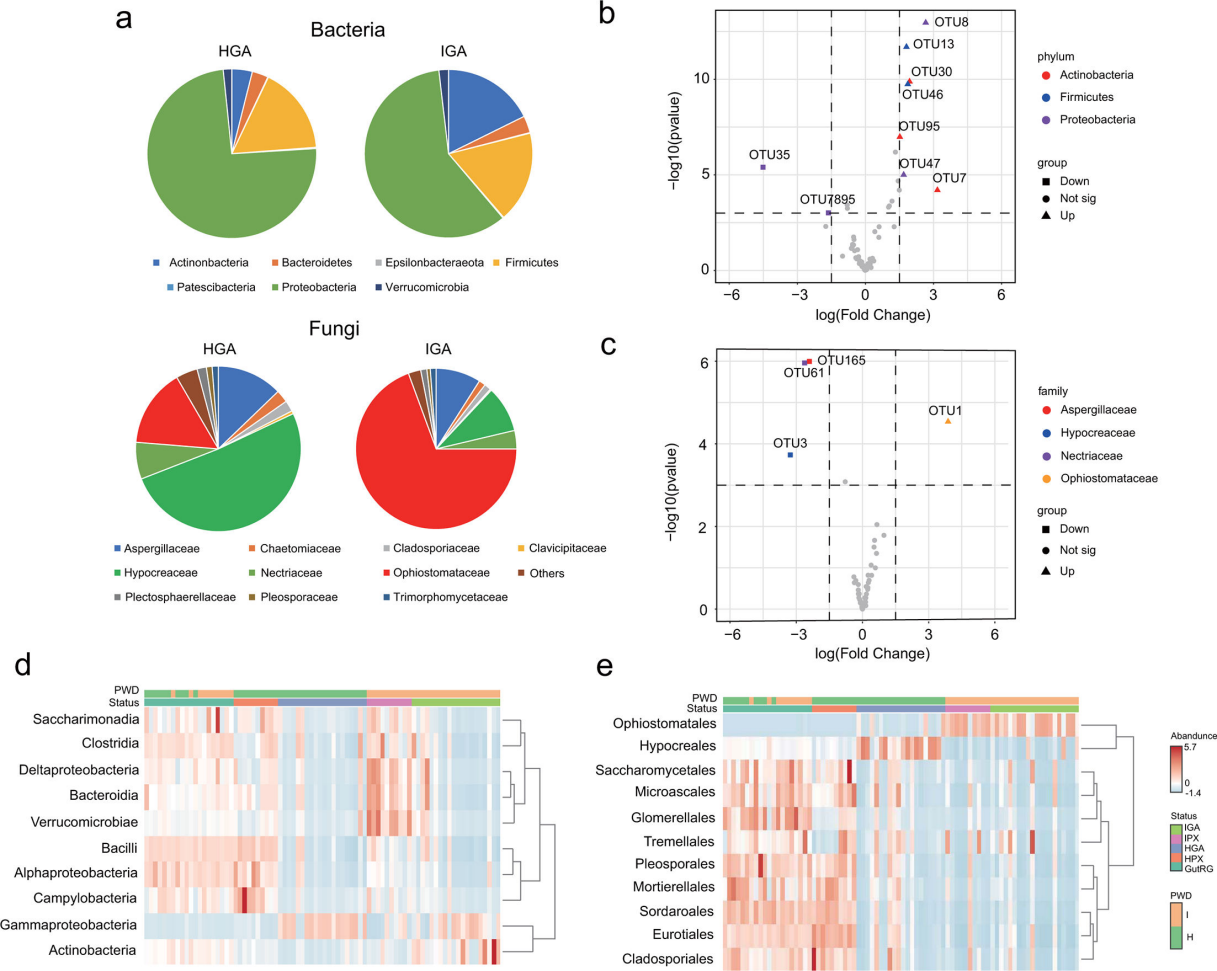
Core microbiome and enriched beneficial taxa across. (**a**) Taxonomic composition of the core microbiome taxa at bacterial phylum and fungal family levels across development process in both healthy and diseased treatments. (**b and c**) Volcano plot illustrating the enrichment and depletion patterns of bacterial (above) and fungal (bottom) core microbiomes in the diseased galleries compared with the healthy ones. The symbols correspond to PWD disease-enriched (triangle) and PWD-depleted (square) OTUs. (**d and e**) Heatmap of the core microbiome at bacterial phylum (**d**) and fungal (**e**) family level, respectively. PWD encompasses healthy (H) and disease infected (I) treatment, while status has GutRG, HPX, HGA, IPX, and IGA.

We identified 60 core bacterial taxa and 48 core fungal taxa present in both healthy and PWD-infected communities (Dataset S4 and S5). The composition of the core microbiome exhibited divergence between healthy and PWD-infected communities. For bacteria, the most abundant taxa at the phylum were Proteobacteria, Firmicutes, and Actinobacteria, with differing relative abundances between healthy (74.3%, 16.7%, and 3.9%, respectively) and PWD-infected (59.3%, 17.6%, and 17.7%, respectively) communities (Fig. 5a). Regarding fungi, the predominant taxa at the family level were Hypocreaceae (51.1%), Ophiostomataceae (15.3%), Aspergillaceae (12.8%), and Nectriaceae (7.2%) in healthy community, while the major taxa were Ophiostomataceae (69.4%), Hypocreaceae (9.3%), Aspergillaceae (9.0%), and Nectriaceae (3.7%) in PWD-infected communities (Fig. 5a).

To identify the enriched and depleted taxa in the PWD-infected galleries, we conducted a difference analysis between healthy and PWD-infected galleries using the core bacterial and fungal communities, respectively. For bacteria, taxa such as OUT8, OUT13, OTU30, OTU46, OTU95, OUT47, and OTU7 belonging to phylum Actinobacteria and Firmicutes were significantly enriched, while OTU35 and OTU7895 belonging to phylum Proteobacteria were significantly depleted (*P* < 0.001, Fig. 5b; Dataset S4). Meanwhile, OUT1 belonging to family Ophiostomataceae was significantly enriched in fungal community, but OUT3, OTU61, and OTU165 were significantly depleted (*P* < 0.001, Fig. 5b; Dataset S5). The heatmap of core microbiome also revealed that the order Gammaproteobacteria and Actinobacteria were most abundant in assembled galleries for bacteria (Fig. 5d). For fungi, the class Ophiostomatales was the most prevalent taxa in PWD-infected assembled galleries, while Hypocreates was the dominant taxa in healthy assembled galleries. Moreover, the relative abundance of these two taxa had opposite directions, indicating their negative correlation (Fig. 5e).

## DISCUSSION

### Effects of PWD on the diversity and functions of host pine microbiome

Investigating the response of the host plant and its associated microbiomes to plant disease is crucial for advancing the co-evolutionary theory of plant-microbiome interactions (43). Our study demonstrated that PWD significantly affects the bacterial and fungal communities in both the sourced xylem and phloem in the assembled galleries. Changes in composition and diversity induced by the pathogen have been reported in many studies (37, 38, 44), but some results exhibit a different tendency of the diversity changes. For example, previous studies revealed that diseased pine trees harbor many more microbes than that inhealthy trees (37, 38), but our results conversely showed that microbial diversity in diseased trees was significantly lower than that in healthy trees, except for bacterial diversity in phloem. The inconsistent results between our and previous ones might be due to the differences in host plant species or parts, geographies, or pathogen infection time (44, 45). Additionally, the diversity of the bacterial community in both healthy and diseased trees showed a significant decrease over the development time of vector insects, while the diversity of fungal communities decreased in healthy galleries but had no change in diseased galleries, suggesting the existence of the microbial filtration during the microbiome assembly process. The unchanged diversity in the fungal community of diseased gallery indicated that prior filtration might have occurred before sampling in the diseased trees, which is supported by the similar composition between sourced logs (xylem and phloem) and the galleries, both of which were dominated by Ophiostomataceae. The high similarity of the lowest value of Shannon diversity between healthy and diseased galleries in both bacterial and fungal communities further support this speculation.

### Changes in the assembly process of gallery microbiome

Metacommunity ecology methods have garnered increasing popularity in microbiome studies (33, 46). Similarly, we employed both the neutral community model and the null model to investigate the assembly processes of the gallery microbiome. The results demonstrated that the deterministic processes dominated the microbial community assembly in both healthy and diseased galleries, consistent with patterns found in previous studies (33, 39). Furthermore, the results from the neutral community model showed that the diseased gallery microbiome exhibited a higher quality of fit and accounted for much higher proportion of the cumulative relative abundances of the OTUs below prediction compared to the healthy gallery microbiome, in both bacterial and fungal communities. Additionally, the negative correlations of microbial interactions of the co-occurrence networks, both inter-kingdom and intra-kingdom, decreased from the source pine trees to the assembled galleries in both healthy and diseased treatments. However, the extent of the changes in healthy treatment was much higher than that in diseased treatment, indicating a divergence in stability of species interactions. This divergence may be due to mutually negative interactions, signifying ecological competition, which may enhance microbiome stability by mitigating the destabilizing impacts of cooperation (47). Moreover, an increasing body of studies and ecological observations suggest that fungal communities are generally more sensitive to environmental changes compared to bacterial communities (48–51). Our results also supported this argument. First, PERMANOVA analysis indicated that PWD had a higher impact on fungal communities than bacterial communities, as evidenced by much higher divergence in fungal communities between healthy and diseased trees induced by PWD. Additionally, bacterial networks in both healthy and diseased galleries were characterized by a higher proportion of negative correlations than those in the fungal networks. Collectively, these results suggested that the assembly of gallery microbiomes was strongly influenced by pathogen invasion, consistent with previous reports (48, 52, 53). The observed changes may result from breakdown of the plant immune system or a stress response by the defense system, warranting further investigation (54).

As our experiments were conducted in a controlled condition, where the only difference between healthy and diseased gallery microbiomes lay in the local regional species pool (i.e., source microbiota) induced by PWD, we hypothesized that the priority effects, host selection and filtration, and microbial interactions collectively contribute to the divergent assembly processes induced by PWD between healthy and diseased gallery microbiomes. This hypothesis is based on the following points: (i) Changes in local regional species pool exert a priority effect on the subsequent assembly process. Priority effect refers to the phenomenon where the effect of species on one another depends on the particular order and timing in which different species join communities (55, 56). This effect can lead to significant changes in the structure and function of microbial communities (57), as their effects are magnified over time and space via population growth and interactions (58). Source tracking analysis supported this finding, showing an increased proportion of microbes originated from pine trees (phloem and xylem) over time in the diseased bacterial and fungal communities; (ii) The decrease in diversity of the assembled gallery microbiome suggested that host insects might play an important role in selecting and filtering core microbiome. Vector beetle larva likely selectively recruit and maintain specific microbial taxa within their galleries based on their physiology, behavior, and immune system, as well as the chemical compounds derived from the wood substrate or secreted by the insects. (iii) Cooperative and competitive interactions among microbial species can influence species sorting and the community assembly process (59). Therefore, the divergence of changes in negative interactions of co-occurrence networks, indicating ecological competition, suggested that microbial interactions might shape the final composition of gallery microbiome. A limitation of this result is the lack of generalizability validation in different regions. However, concerning the taxa enriched by PWN and their selective acquisition by deterministic factors, our findings align with previous research (33, 35), which involved sampling across different geographical locations. This suggests that our findings may indeed be applicable across different regions to some extent.

### Enrichment of beneficial microbiome in gallery enhances the fitness of vector beetle

The gallery serves as a critical zone for interactions between wood-boring insects and their associated microbiome. It is a hidden and enclosed space where offspring develop from egg to larva to pupa. These unique features allow the gallery microbiome to undergo simple succession over development, controlled by a few variable factors such as local species pool, host selection and filtration, and microbial species interactions (31, 32). This setup increases the fidelity of transmission of keystone microbes, ultimately forming a core beneficial microbiome. The data from this study also supported this perspective. For instance, the dominant taxa, such as Ophiostomatoid fungi, are horizontally transmitted from the source trees and undergo a filtration process, as evidenced by their significant increase in abundance in diseased galleries. Another intriguing finding is the opposite expression pattern observed between beneficial microbes enriched in galleries and some antagonistic microbes. For instance, an antagonistic relationship between Ophiostomatales and Hypocreales in fungi, regarding their relative abundance, has been extensively studied (36, 60). This relationship is often governed by chemical compounds. For example, Hypocreales, such as *Trichoderma*, have exhibited significant antagonism towards Ophiostomatoid fungi by secreting 6-Pentyl-α-pyrone, positioning their potential as biological control agents against sap stain fungi (60). This suggests that microbial interactions mediated by chemicals could serve as a potent driving force behind the recruitment of a beneficial microbiome.

Deciphering the core taxa and their correlations with the host insects is critical for understanding how the microbiome affects insect adaptation (61, 62). In this study, several potential beneficial bacteria, such as Actinobacteria and Firmicutes, and fungi like Ophiostomataceae, were enriched in diseased galleries and identified as the core taxa (present in all samples). Actinobacteria and Firmicutes have been widely reported as the symbiotic bacteria of host insects, providing nutrition supplementation and protection against pathogens (3, 63). Ophiostomatoid fungi often form symbiotic associations with bark or wood- boring insects, assisting in the dispersal of their inocula (64). For example, *Sporothrix* sp. 1, an Ophiostomatoid fungus prevalent in the PWD system, induces the production of active compound diacetone alcohol (DAA) in the pine xylem, enhancing the development and survival rate of arthropods such as *M. alternatus* (35).

Wood-boring beetles exhibit an oviposition preference for weakened trees infected with pathogens (40). The galleries of trees infected with PWD exhibit restricted nutrient availability and harbor abundant potential pathogens, conditions that might be harmful to beetle offspring (41). However, our results revealed the selection and enrichment of potential beneficial microbes during the assembly process, leading to the corresponding promotion of larval beetle development in diseased galleries. Based on these findings, we hypothesize that the beneficial microbiome may enhance the fitness of vector beetles by overcoming nutritional limitations or defending against the pathogens, aligning with findings from previous studies (6, 42). However, since our primary focus was to explore the microbiome assembly process at the community level, we have not yet conducted functional verification of specific beneficial microbes. Further investigations are warranted in this regard.

In conclusion, we demonstrated that PWD could influence the assembly process of gallery microbiome, leading to changes in the taxonomic composition of bacterial and fungal communities in both source trees and assembled galleries. Priority effects, host selection and filtration, and microbial interactions emerge as potentially crucial drivers in selecting, filtering, and enriching beneficial microbial taxa within the diseased galleries, thereby promoting the development of the larval vector insect. Our findings shed new light on the understanding of assembly mechanisms of microbial communities within galleries, which represent a typical hidden and enclosed microhabitat. Moreover, by investigating the assembly process along a development time series, we offer valuable insights into the dynamics of microbiome establishment in these environments. Furthermore, this study also enhances the comprehensive understanding of the prevalence of PWD and encourages the development of novel strategies of pest and pathogen management based on microbiome manipulation.

## MATERIALS AND METHODS

### Sampling

In our laboratory-controlled comparison experiment, depicted in Fig. 1a, we aimed to compare the sources and assembly processes of gallery microbiomes between healthy and diseased trees. For this purpose, we categorized the samples into three main groups: (i) source beetle samples: These include samples from the gut (Gut) and reproductive glands (RG) of the female beetles; (ii) source pine samples: These samples comprise the xylem and phloem extracted from the pine tree logs. Together with the source beetle samples, they are utilized for source tracking and transmission analysis; (iii) assembly gallery samples: These samples were collected over a developmental time series from the galleries and used for analyzing the microbial dynamics and functional properties (Fig. 1a).

#### Source beetle samples

Five pairs of male and female *M. alternatus* beetles were reared on the fresh pine branches. After 10 days, a portion of female beetles was selected for source beetle sample collection after removing the body surface microorganisms with 75% alcohol. Between 3 and 7 samples of gut and reproductive glands were dissected from the mated female *M. alternatus* beetles using sterile tweezers. Four to six individuals were pooled as one sample, resulting in a total of 14 gut and 6 reproductive gland samples as the source beetle samples. The remaining beetles were transferred to a rearing box containing logs (8–10 cm in diameter and 50 cm in length) sourced either from healthy or PWD-infected trees for laboratory-controlled comparison experiments.

#### Source pine samples

Pine wilt disease (PWD) early-infected and healthy pine trees (height: 5–7 m) were collected from Chizhou (30°32′45.209″N, 117°46′48.069″E), in Anhui Province, for the source pine sampling. The early-infected trees defined as those exhibiting slightly yellowed needles with decreased resin secretion and growth rate, while healthy pine trees displayed dark green needles, normal resin secretion, and vigorous growth (65). The selected trees were cut into logs without insect egg-laying holes (5–6 cm in diameter and 40 cm in length), and the cross-sections at both ends were waxed before storing them in a sterile 4°C cooling room. To confirm the logs' health status, the presence of pinewood nematode was assessed using the Baermann funnel method on three randomly selected logs from each tree. Xylem was collected using a sterile electric drill, while phloem was sampled using a sterile scalpel and tweezers from the healthy and diseased logs, respectively. Four to five xylem or phloem samples from different logs were pooled as one sample. Finally, five xylem and five phloem samples were prepared as source pine samples for DNA extraction.

#### Assembly gallery samples

After 24 h of the egg-laying experiments, the logs were utilized for hatching and larval development, followed by the collection of assembly gallery samples over developmental time. In brief, the number of oviposition holes in each log was counted, and then they were placed into separate germ-free boxes, maintaining darkness at 25°C with 37% humidity in a climate chamber. To prevent mutual contamination, the healthy and PWD-infected logs were kept in different boxes. The eggs hatched approximately one week later, initiating their larval development. After 10, 15, 25, and 40 days, five logs from either healthy and diseased treatment were debarked or split to collect larva for survival rate calculation and weight measurement. Additionally, galleries were dissected for DNA extraction. Sawdust from the surface of three to five pupal chambers across multiple logs was scraped using a sterile scalpel and tweezers and pooled as one sample, resulting in 80 gallery samples in total from both healthy and diseased logs. All samples were stored at −80°C and utilized for genomic DNA extraction.

### Survival rate and body weight measurement

The egg-laying holes were counted for each log when the logs taken out from egg-laying experiment. At each sampling time point, five logs from each treatment were debarked or split to collect larva when sampling the gallery. The number of live larvae was counted, and the survival rate was calculated using the following formula: Survival rate = number of live larvae/number of egg-laying holes. Additionally, the weight of each larval beetle was measured using a scale with 0.1 mg precision.

### Genomic DNA extraction and Illumina sequencing

The total genomic DNA of all samples was extracted using the TGuide S96 Magnetic Soil/Stool DNA Kit (Tiangen Biotech Co., Ltd., Beijing) following the manufacturer’s instructions. The V3–V4 region of the bacterial 16S rRNA gene and the fungal nuclear ribosomal internal transcribed spacer region ITS1 region were polymerase chain reaction (PCR) amplified (95°C for 5 min followed by 25 cycles of 95°C for 30 s, 50°C for 30 s, and 72°C for 40 s, with a final extension at 72°C for 7 min) with barcoded primers 338F/806R and ITS1F/ITS2R, respectively (Table S1). The PCR products were purified with Agencourt AMPure XP Beads (Beckman Coulter, Indianapolis, IN) and quantified using the Qubit dsDNA HS Assay Kit and Qubit 4.0 Fluorometer (Invitrogen, Thermo Fisher Scientific, OR, USA). The quality-checked amplicon libraries were pooled to an equimolar concentration and sequenced by Illumina Novaseq 6000 sequencing platform by Biomarker Technologies (Beijing, China).

### Amplicon sequencing data processing

The bacterial 16S rRNA gene and fungal ITS sequences were processed with the assistance of the BMK Cloud (Biomarker Technologies Co., Ltd., Beijing, China). Specifically, raw data were filtered using Trimmomatic v0.33 (66). Primers were identified and removed using Cutadapt 1.9.1 software (67). Paired-end reads were then assembled by Usearch v10.0 (68) and subjected to chimera removal using UCHIME v8.1 (69). The high-quality reads generated from these steps were utilized in the subsequent analysis.

Unique sequences were clustered into operational taxonomic units (OTUs) at a threshold of 97% similarity using Usearch v10.0, and OTUs with abundances <0.005% were filtered out. Taxonomy annotation of the OTUs was performed based on the Naive Bayes classifier in QIIME2 (70) using the SILVA database (release 132, http://www.arb-silva.de) (69) with a confidence threshold of 70%. The community composition of each sample was determined at each taxonomic level (phylum, class, order, family, genus, and species). To minimize potential bias, OTUs represented by fewer than two sequences or classified as mitochondria or chloroplasts were excluded from further analysis.

### Statistical analyses

The composition of bacterial and fungal communities of dominant families with relative abundances ≥1.0% in both source and assembled samples from healthy and diseased logs were calculated and visualized using BMKCloud (www.biocloud.net), respectively. The bacterial and fungal OTU tables were rarefied to 1,608 and 1,369 sequences per sample for alpha diversity index estimates, respectively. Alpha diversity was estimated using Shannon index and calculated by the QIIME2. Differences among samples of each time point from healthy and diseased logs were tested using the Wilcoxon rank-sum test. Non-metric multi-dimensional scaling (NMDS) was performed using Bray–Curtis distance matrices for beta-diversity analysis. To ascertain the extent of the effects of different factors (PWD, compartment and time) on community dissimilarity, permutational multivariate analysis of variance (PERMANOVA) statistical tests were performed using the “adonis” function of vegan package in vegan R package, with 999 permutations and using Bray–Curtis distance matrix as an input (71).

LEfSe (68) was employed to identify significant taxonomic differences between healthy and diseased galleries. A logarithmic LDA score of 4.0 was set as the threshold for discriminative features, and the taxonomic level was selected from family to genus.

We used neutral and null models to examine the assembly process of gallery microbiomes. The Sloan NCM was employed to evaluate the significance of neutral process in the assembly of bacterial and fungal communities within healthy and disease galleries, using R code as described by Burns et al. (72). In essence, the model predicts that species more abundant in the metacommunity are more likely to be widespread, as they have a higher chance of dispersing by chance. Conversely, less abundant species are more susceptible to extinction due to ecological drift (73). The metacommunity of galley microbiomes was separately analyzed using the bacterial and fungal data sets from both healthy and diseased logs, respectively. OTUs were categorized into three partitions based on the 95% confidence interval around the neutral model prediction: above prediction (occurring more frequently and/or at greater abundance than predicted by the neutral model), below prediction (occurring less frequently and/or at lower abundance than predicted), and neutral distribution (falling within the prediction range). The coefficient of determination (*R*^2^) reflects the goodness of fit of the neutral model. Furthermore, the MST was employed to quantify the relative importance of deterministic and stochastic processes in microbial community assembly. An MST value <50% indicates deterministic dominance, while an MST value >50% suggests stochastic dominance. The MST analyses were conducted using the “nst” package, based on Bray–Curtis distance (74). Furthermore, we employed the null modeling approach to assess phylogenetic patterns by computing the βNTI for sample pairs, following the methodology outlined in Stegen et al. (20), This analysis utilized the R package “icamp,” as detailed by Ning et al. (75). Sample pairs exhibiting |βNTI| >2 are anticipated to arise from deterministic processes, whereas values of |βNTI| <2 suggest weak selection pressure, indicating that community assembly is likely influenced by stochastic processes (20, 75).

FEAST, based on the Bayesian approach (76), was employed to estimate the sources of microbial communities within galleries across different sampling time points. In this analysis, the source samples included gut and reproductive gland samples from female beetles, as well as xylem and phloem samples from the egg-free logs. These source samples were designated as “source,” while the gallery assembly samples collected at various time points were set as “sinks.

The data obtained from the samples of the healthy (HPX) and PWD-infected (IPX) xylem and phloem, as well as the samples of the healthy (HGA) and PWD-infected (IGA) galleries, were grouped for co-occurrence network construction, respectively. Network construction was performed using the “ggClusterNet” package in R. For the interkingdom network, the “corBionetwork” function was used to build the network module, and the network module was calculated based on the “cluster fast greedy” algorithm, with a threshold of *R* = 0.05 and *P* value = 0.75. For the intrakingdom network, the “network” function with default parameter was employed. In each network, nodes represent individual microbial OTUs, and edges represent pairwise correlations between nodes, indicating biologically or biochemically meaningful interactions between microbial taxa. Topological characteristics, including the numbers of nodes, number of edges (positive and negative correlations), average path length, and clustering coefficient, were calculated. The complexity of networks was evaluated using the number of nodes and edges as well as the average path length and clustering coefficient (77), and the network stability was measured by the proportion of negative or positive correlations and the modularity (47).

The core microbiome of healthy and diseased galleries was defined as OTUs present in 100% samples from gut, reproductive gland, xylem, phloem, and gallery of healthy and diseased logs, respectively. For differential abundance and enrichment analysis of OTUs in diseased galleries compared to healthy ones, EdgeR’s generalized linear model (GLM) approach (78) was used for differential abundance and enrichment analysis of OTUs in diseased galleries compared to healthy ones, using a trimmed mean of *M* values (TMM) normalization method and a threshold of significance at *P* < 0.001 in “edgeR” package. MicrobiomeAnalyst (https://www.microbiomeanalyst.ca/) (79) was employed to construct the heatmap of the core microbiome at the taxonomic level of class and order for bacterial and fungal communities, respectively.

## Data Availability

The raw data were deposited in the Genome Sequence Archive (GSA) in National Genomics Data Center under the accession number CRA016098 for 16S rRNA and CRA016100 for ITS, respectively. All other data supporting the conclusions of this article are included within the article and its additional files.

## SUPPLEMENTAL MATERIAL

Dataset S1-S5 spectrum.01412-24-s0001.xlsx The datasets supporting the results.

Supplemental figures and tables spectrum.01412-24-s0002.docx Fig. S1-S3; Tables S1-S6.

ASM does not own the copyrights to Supplemental Material that may be linked to, or accessed through, an article. The authors have granted ASM a non-exclusive, world-wide license to publish the Supplemental Material files. Please contact the corresponding author directly for reuse.
