# Supplementary material for: Pinewood nematode induced changes in the assembly process of gallery microbiomes benefit its vector beetle’s development
Source: Microbiol Spectr. 2024 Sep 11;12(10):e01412-24. doi: 10.1128/spectrum.01412-24 (PMC11448173; doi:10.1128/spectrum.01412-24)
Supplement: Supplemental figures and tables — Fig. S1-S3; Tables S1-S6. [file spectrum.01412-24-s0002.docx]

**Pathogen induced changes in the assembly process of gallery microbiomes benefit its vector beetle’s development**

Bin Zhang^1^, Yafei Ma^1^, Wenzhao Duan^1^, Qi Fan^1^, Jianghua Sun^1, 2*^

^1^ College of Life Science/Hebei Basic Science Center for Biotic Interactions, Institute of Life Science and Green Development, Hebei University, Baoding, 071002, China

^2^ State Key Laboratory of Integrated Management of Pest Insects and Rodents, Institute of Zoology, Chinese Academy of Sciences, Beijing, China

Correspondence author:

Jianghua Sun, [sunjh@hbu.edu.cn](mailto:sunjh@hbu.edu.cn)

**Figure S1** **β-nearest taxon index (βNTI) of gallery microbiome assembly across both bacterial (a) and fungal (b) communities.**


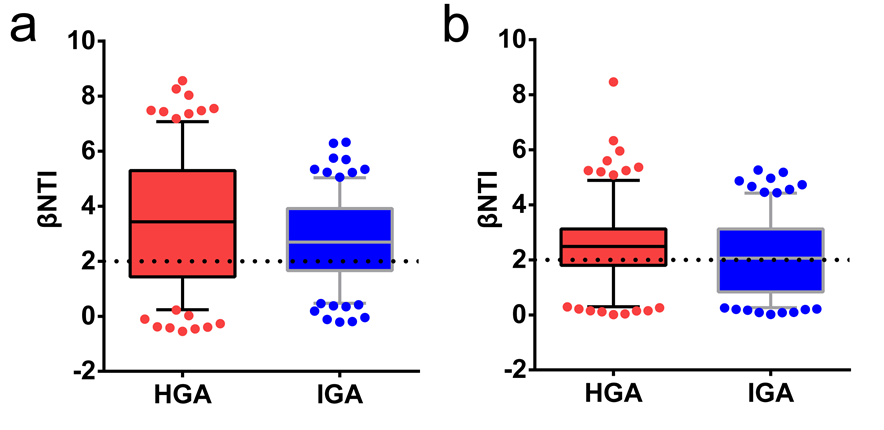


**Figure S2** **Bacterial-bacterial intrakindom co-occurrence networks and the parameters. a**. Interkingdom co-occurrence networks. **b**. Comparison of the values of average path length and clustering coefficient for source (PX) and gallery (GA) samples in both healthy and PWD-infected treatments. **c**. The number of bacterial-bacterial correlations in the healthy and diseased networks.


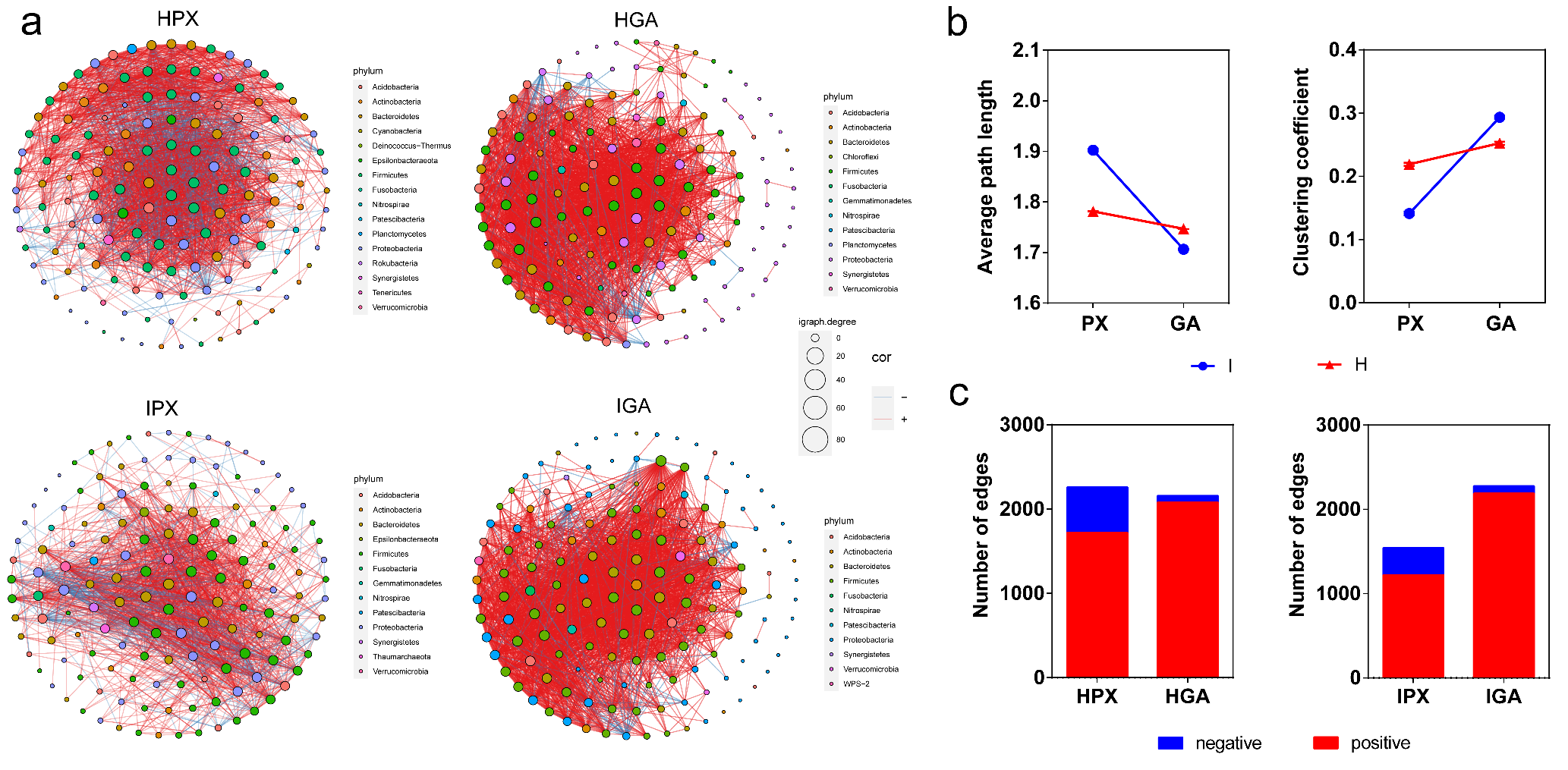


**Figure S3** **Fungal-fungal intrakindom co-occurrence networks and the parameters. a**. Interkingdom co-occurrence networks. **b**. Comparison of the values of average path length and clustering coefficient for source (PX) and gallery (GA) samples in both healthy (H) and PWD-infected (I) treatments. **c**. The number of fungal-fungal correlations in the healthy and diseased networks.


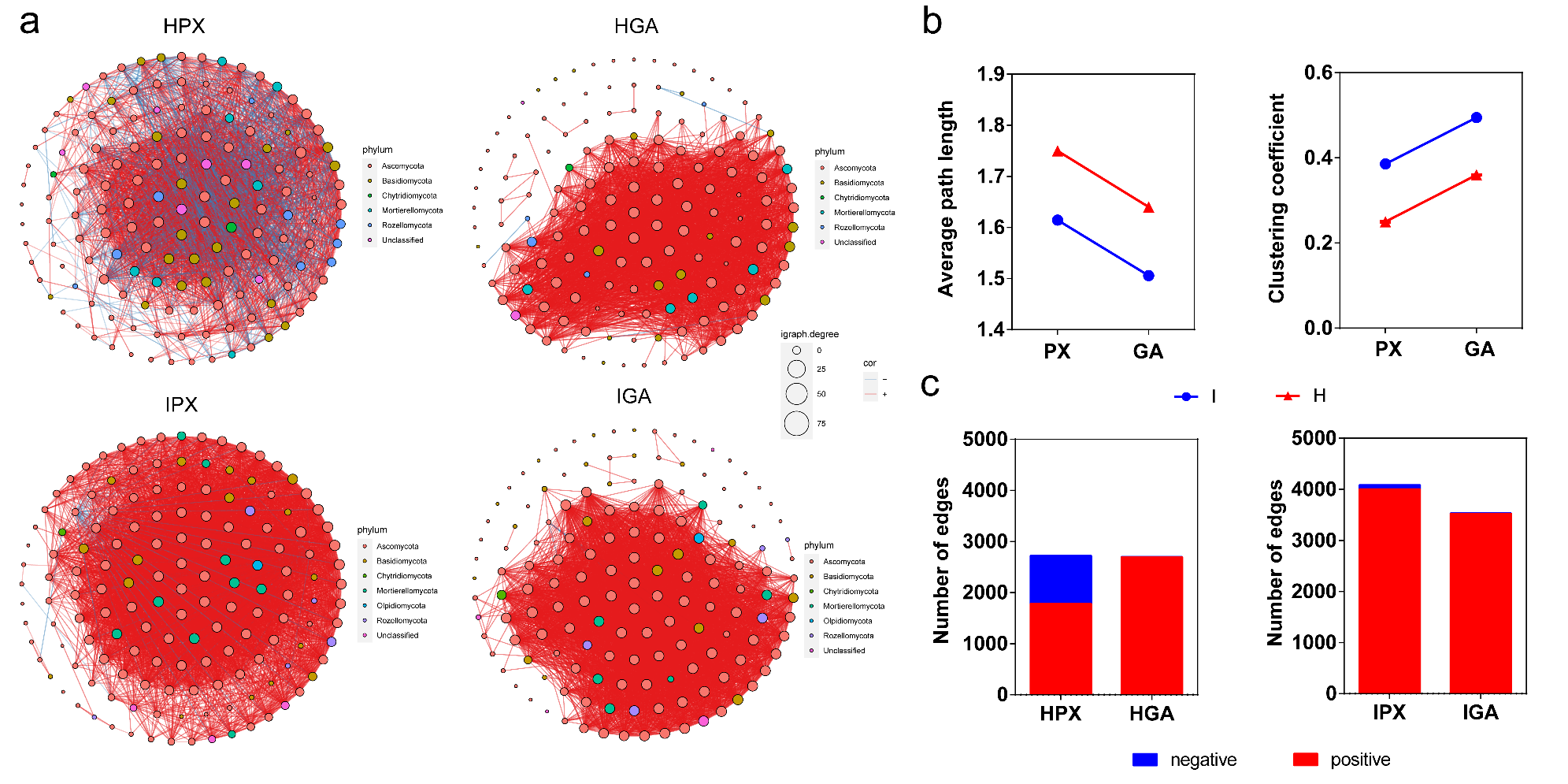


**Table S1** Primers used in this study.

| Primer | sequence (5'-3') | Length (bp) | Target | Reference |
| --- | --- | --- | --- | --- |
| 338F | ACTCCTACGGGAGGCAGCA | 300-500 bp | Bacterial V3-V4 region of 16S rRNA gene | Dennis et al (2013) |
| 806R | GGACTACHVGGGTWTCTAAT |  |  |  |
| ITS1F | CTTGGTCATTTAGAGGAAGTAA | / | Fungal ITS1 gene region | Admas et al (2013) |
| ITS2R | GCTGCGTTCTTCATCGATGC |  |  |  |

**Table S2** Relative abundance of top 10 families in bacterial communities in both source and assembly samples.

| Families | HGut | HRG | HP | HX | H10GA | H15GA | H25GA | H40GA | IGut | IRG | IP | IX | I10GA | I15GA | I25GA | I40GA |
| --- | --- | --- | --- | --- | --- | --- | --- | --- | --- | --- | --- | --- | --- | --- | --- | --- |
| Enterobacteriaceae | 0.203 | 0.248 | 0.056 | 0.092 | 0.335 | 0.277 | 0.262 | 0.273 | 0.272 | 0.244 | 0.114 | 0.115 | 0.202 | 0.268 | 0.280 | 0.183 |
| Xanthomonadaceae | 0.004 | 0.004 | 0.002 | 0.005 | 0.216 | 0.206 | 0.275 | 0.215 | 0.004 | 0.004 | 0.041 | 0.004 | 0.083 | 0.137 | 0.258 | 0.129 |
| Rhodanobacteraceae | 0.002 | 0.001 | 0.002 | 0.002 | 0.010 | 0.037 | 0.027 | 0.059 | 0.002 | 0.002 | 0.042 | 0.146 | 0.070 | 0.211 | 0.085 | 0.207 |
| Ruminococcaceae | 0.062 | 0.057 | 0.128 | 0.073 | 0.032 | 0.036 | 0.033 | 0.023 | 0.056 | 0.063 | 0.070 | 0.052 | 0.051 | 0.022 | 0.010 | 0.014 |
| Lactobacillaceae | 0.049 | 0.048 | 0.114 | 0.094 | 0.037 | 0.044 | 0.036 | 0.011 | 0.047 | 0.053 | 0.044 | 0.037 | 0.051 | 0.018 | 0.009 | 0.011 |
| Lachnospiraceae | 0.047 | 0.065 | 0.035 | 0.060 | 0.025 | 0.029 | 0.027 | 0.025 | 0.046 | 0.055 | 0.076 | 0.057 | 0.055 | 0.018 | 0.007 | 0.012 |
| Burkholderiaceae | 0.030 | 0.033 | 0.090 | 0.027 | 0.046 | 0.017 | 0.014 | 0.051 | 0.036 | 0.032 | 0.022 | 0.036 | 0.027 | 0.033 | 0.039 | 0.033 |
| Sphingomonadaceae | 0.013 | 0.012 | 0.021 | 0.026 | 0.013 | 0.014 | 0.025 | 0.071 | 0.012 | 0.012 | 0.032 | 0.012 | 0.016 | 0.070 | 0.046 | 0.057 |
| Muribaculaceae | 0.028 | 0.033 | 0.035 | 0.045 | 0.020 | 0.021 | 0.017 | 0.012 | 0.024 | 0.024 | 0.046 | 0.038 | 0.034 | 0.011 | 0.004 | 0.005 |
| Streptococcaceae | 0.031 | 0.033 | 0.009 | 0.045 | 0.021 | 0.024 | 0.019 | 0.008 | 0.031 | 0.030 | 0.021 | 0.015 | 0.022 | 0.011 | 0.005 | 0.007 |
| Others | 0.531 | 0.466 | 0.506 | 0.530 | 0.243 | 0.295 | 0.265 | 0.253 | 0.470 | 0.481 | 0.492 | 0.488 | 0.390 | 0.201 | 0.257 | 0.342 |
| Sum of Top 10 families | 0.469 | 0.534 | 0.494 | 0.470 | 0.757 | 0.705 | 0.735 | 0.747 | 0.530 | 0.519 | 0.508 | 0.512 | 0.610 | 0.799 | 0.743 | 0.658 |

* The cells in blue represent source samples

# The cells in yellow represent assembly samples

**Table S3** Relative abundance of top 10 families in fungal communities in both source and assembly samples.

| Families | HGut | HRG | HP | HX | H10GA | H15GA | H25GA | H40GA | IGut | IRG | IP | IX | I10GA | I15GA | I25GA | I40GA |
| --- | --- | --- | --- | --- | --- | --- | --- | --- | --- | --- | --- | --- | --- | --- | --- | --- |
| Ophiostomataceae | 0.002 | 0.002 | 0.001 | 0.006 | 0.141 | 0.033 | 0.076 | 0.221 | 0.002 | 0.008 | 0.732 | 0.549 | 0.429 | 0.551 | 0.629 | 0.366 |
| Aspergillaceae | 0.174 | 0.211 | 0.222 | 0.248 | 0.099 | 0.103 | 0.112 | 0.047 | 0.180 | 0.189 | 0.042 | 0.076 | 0.084 | 0.056 | 0.011 | 0.011 |
| Hypocreaceae | 0.007 | 0.004 | 0.006 | 0.006 | 0.381 | 0.260 | 0.372 | 0.257 | 0.006 | 0.009 | 0.019 | 0.017 | 0.035 | 0.097 | 0.017 | 0.061 |
| Nectriaceae | 0.102 | 0.107 | 0.125 | 0.101 | 0.031 | 0.067 | 0.128 | 0.175 | 0.111 | 0.112 | 0.014 | 0.037 | 0.034 | 0.015 | 0.004 | 0.004 |
| Chaetomiaceae | 0.081 | 0.079 | 0.069 | 0.068 | 0.020 | 0.024 | 0.018 | 0.010 | 0.076 | 0.078 | 0.012 | 0.026 | 0.023 | 0.011 | 0.003 | 0.003 |
| Trichocomaceae | 0.013 | 0.014 | 0.012 | 0.017 | 0.007 | 0.011 | 0.006 | 0.004 | 0.013 | 0.017 | 0.034 | 0.087 | 0.021 | 0.008 | 0.070 | 0.128 |
| Phacidiaceae | 0.000 | 0.000 | 0.000 | 0.000 | 0.001 | 0.000 | 0.000 | 0.000 | 0.000 | 0.000 | 0.026 | 0.000 | 0.063 | 0.140 | 0.097 | 0.077 |
| Plectosphaerellaceae | 0.047 | 0.075 | 0.012 | 0.027 | 0.009 | 0.010 | 0.009 | 0.006 | 0.038 | 0.043 | 0.004 | 0.015 | 0.012 | 0.006 | 0.001 | 0.001 |
| Debaryomycetaceae | 0.021 | 0.001 | 0.004 | 0.002 | 0.107 | 0.147 | 0.011 | 0.002 | 0.004 | 0.003 | 0.000 | 0.002 | 0.000 | 0.000 | 0.001 | 0.003 |
| Cantharellales_fam_  Incertae_sedis | 0.001 | 0.000 | 0.000 | 0.000 | 0.000 | 0.000 | 0.000 | 0.000 | 0.000 | 0.001 | 0.000 | 0.001 | 0.105 | 0.007 | 0.084 | 0.096 |
| Others | 0.385 | 0.358 | 0.393 | 0.344 | 0.156 | 0.279 | 0.224 | 0.252 | 0.399 | 0.399 | 0.085 | 0.144 | 0.142 | 0.073 | 0.059 | 0.153 |
| Unclassified | 0.166 | 0.149 | 0.157 | 0.182 | 0.050 | 0.064 | 0.042 | 0.026 | 0.171 | 0.139 | 0.031 | 0.046 | 0.051 | 0.036 | 0.024 | 0.096 |
| Sum of Top 10 families | 0.449* | 0.493 | 0.450 | 0.474 | 0.794# | 0.657 | 0.733 | 0.722 | 0.430 | 0.462 | 0.884 | 0.811 | 0.807 | 0.891 | 0.917 | 0.751 |

* The cells in blue represent source samples

# The cells in yellow represent assembly samples

**Table S4** PERMANOVA by adonis of all bacterial 16S and fungal ITS samples. PERMANOVA analysis using the Bray Curtis distances for compartment, PWD, and time in beta diversity.

| Microbial communities | Variables | Df^a^ | | SumsOfSqs^b^ | | MeanSqs^c^ | F.Model | | *R*^2^ | Pr(>F)^d^ |
| --- | --- | --- | --- | --- | --- | --- | --- | --- | --- | --- |
| Bacterial community | Compartment | 4 | 2.748 | | 0.687 | | 3.419 | 0.154 | | 0.001 *** |
|  | time | 4 | 1.530 | | 0.382 | | 1.761 | 0.086 | | 0.001 *** |
|  | PWD | 1 | 1.139 | | 1.139 | | 5.090 | 0.081 | | 0.002 ** |
| Fungal community | Compartment | 4 | 4.449 | | 1.112 | | 4.526 | 0.194 | | 0.001 *** |
|  | time | 4 | 3.858 | | 0.964 | | 3.802 | 0.169 | | 0.001 *** |
|  | PWD | 1 | 3.694 | | 3.694 | | 15.541 | 0.211 | | 0.001 *** |

^a^ degree of freedom, ^b^ sum of squares, ^c^ mean sum of squares, ^d^ p-values are based on 999 permutations with subsequent Bonferroni correction.

**Table S5** The cumulative relative abundance of OTUs in neutral, above and below prediction using neutral community model (NCM)

|  | | HGA | | IGA | |
| --- | --- | --- | --- | --- | --- |
|  |  | Relative abundance | Percentage | Relative abundance | Percentage |
| Bacterial communities | Neutral | 41711 | 0.036 | 173623 | 0.112 |
|  | Above | 749413 | 0.639 | 1335986 | 0.858 |
|  | Below | 382353 | 0.326 | 47269 | 0.030 |
| Fungal communities | Neutral | 41711 | 0.036 | 173623 | 0.112 |
|  | Above | 749413 | 0.639 | 1335986 | 0.858 |
|  | Below | 382353 | 0.326 | 47269 | 0.030 |

|  | Parameters | Healthy_source_tree | Healthy_GA | Infected_source_tree | Infected_GA |
| --- | --- | --- | --- | --- | --- |
| Bacteria-Fungi interkindom networks | num.edges | 770 | 438 | 72 | 44 |
|  | num.pos.edges | 565 | 432 | 59 | 28 |
|  | num.neg.edges | 205 | 6 | 13 | 16 |
|  | num.vertices | 232 | 144 | 82 | 49 |
|  | average.path.length | 3.079±0.011 | 2.932±0.015 | 5.558±0.563 | 4.703±0.714 |
|  | clustering.coefficient | 0.029±0.004 | 0.043±0.007 | 0.017±0.002 | 0.039±0.003 |
| Bacteria-Bacteria intrakindom networks | num.edges | 2259 | 2157 | 1541 | 2277 |
|  | num.pos.edges | 1721 | 2081 | 1223 | 2188 |
|  | num.neg.edges | 538 | 76 | 318 | 89 |
|  | num.vertices | 144 | 131 | 148 | 125 |
|  | average.path.length | 1.781±0.0003 | 1.747±0.0001 | 1.902±0.003 | 1.706±0.000 |
|  | clustering.coefficient | 0.219±0.003 | 0.253±0.002 | 0.141±0.003 | 0.293±0.002 |
| Fungi-Fungi intrakindom networks | num.edges | 2722 | 2700 | 4084 | 3530 |
|  | num.pos.edges | 1782 | 2696 | 4002 | 3525 |
|  | num.neg.edges | 940 | 4 | 82 | 5 |
|  | num.vertices | 148 | 123 | 146 | 120 |
|  | average.path.length | 1.750±0.000 | 1.640±0.000 | 1.614±0.000 | 1.506±0.000 |
|  | clustering.coefficient | 0.250±0.002 | 0.360±0.002 | 0.386±0.001 | 0.494±0.001 |

**Table S6** The parameters of co-occurrence networks.
